# Novel Mutations Conferring Amoxicillin Resistance in *Helicobacter pylori* in South Korea

**DOI:** 10.3390/antibiotics12040748

**Published:** 2023-04-13

**Authors:** Soon Young Park, Eun Hwa Lee, Dokyun Kim, Young Goo Song, Su Jin Jeong

**Affiliations:** 1 Division of Infectious Diseases, Department of Internal Medicine, Gangnam Severance Hospital, Yonsei University College of Medicine, Seoul 06273, Republic of Korea; 2 Department of Laboratory Medicine and Research Institute of Bacterial Resistance, Yonsei University College of Medicine, Seoul 03722, Republic of Korea; 3 Department of Internal Medicine, Yonsei University College of Medicine, Seoul 03722, Republic of Korea

**Keywords:** *Helicobacter pylori*, antimicrobial susceptibility testing, whole-genome sequencing, single-nucleotide polymorphism, amoxicillin, clarithromycin, resistance

## Abstract

*Helicobacter pylori* is the primary causative agent of gastritis, gastric ulcers, duodenal ulcers, gastric cancer, and peripheral B-cell lymphoma. *H. pylori* eradication often fails due to elevated antibiotic resistance. However, no previous studies have thoroughly examined amoxicillin resistance. Here, the objective was to identify clinical strains of *H. pylori* with amoxicillin resistance and to analyze single-nucleotide polymorphisms (SNPs) associated with amoxicillin resistance. From March 2015 to June 2019, genotypic and phenotypic amoxicillin resistance was analyzed using an E-test and whole-genome sequencing (WGS). Analysis of 368 clinical strains confirmed amoxicillin resistance in 31 strains (resistance rate of 8.7%). The genomes were extracted from nine resistant (<0.125 mg/L) strains, and WGS was performed for genetic analysis. WGS analysis identified SNPs present in *pbp1a*, *pbp2*, *nhaC*, *hofH*, *hofC*, and *hefC* in all nine isolates. Some of these genes may be related to amoxicillin resistance. A total of six SNPs (A69V, V374L, S414R, T503I, A592D, and R435Q) were identified in PBP2 of H-8, the most resistant strain. We predict that these six SNPs are associated with high amoxicillin resistance. Amoxicillin resistance should be considered in the clinical setting for the treatment failure of *H. pylori* eradication.

## 1. Introduction

*Helicobacter pylori* was first discovered in 1983, and *H. pylori* infection is associated with the pathogenesis of gastric ulcers, duodenal ulcers, gastric cancer, and peripheral B-cell lymphoma [1]. After *H. pylori* was first successfully cultured in 1982, numerous studies tried to determine the pathogenesis and treatment of *H. pylori* [1]. A report from 2013 indicated that the *H. pylori* prevalence rate in South Korea was reported to be 54% [2]. Since 1998, triple therapy containing clarithromycin and amoxicillin has been widely used to treat *H. pylori* infection. However, the success rate of *H. pylori* eradication treatment is decreasing owing to an increase in antibiotic resistance [3]. Eradication rates of *H. pylori* infection with a proton-pump inhibitor (PPI)-based triple therapy have reported a success rate of less than 80% worldwide. Clarithromycin resistance has been reported to be 32.3% in the United States and 17.8% in South Korea [4,5]. Such a high resistance rate is associated with treatment failures in patients and, thus, requires clinicians’ attention. In addition, amoxicillin resistance in PPI-based triple therapy has recently shown a rapid increase by approximately 15% [6]. Therefore, it is necessary to develop new strategies to assess antibiotic resistance and select an appropriate treatment regimen [7,8,9]. To prevent treatment failure owing to antibiotic resistance, patient-tailored antimicrobial therapy through antibiotic resistance testing of clinical samples from patients has been proposed [10]. Some studies have reported that eradication therapy optimized for the *H. pylori* strain derived from patient samples is likely to have a higher success rate than that of conventional methods, considering treatment duration and cost [11]. However, because *H. pylori* is difficult to cultivate in a laboratory environment and antibiotic resistance testing requires additional time and cost, customized eradication therapy is difficult to use in real-world clinical practice [10,11].

In the clinical practice, a rapid diagnostic kit using polymerase chain reaction (PCR) is used to rapidly identify single-nucleotide polymorphisms (SNPs) (A2142G and A2143G) which are associated with clarithromycin resistance [12]. However, the PCR method cannot be applied to identify amoxicillin resistance because no specific SNPs have been reported. 

This study’s purpose was to prospectively collect clinical strains, investigate the amoxicillin resistance rate, and identify SNPs associated with amoxicillin resistance using whole-genome sequencing (WGS) to provide a basis for antibiotic resistance testing to aid *H. pylori* eradication therapy.

## 2. Results

This study tested 1952 patient specimens, with 368 *H. pylori* strains isolated using matrix-assisted laser desorption/ionization-time-of-fight mass spectrometry (MALDI-TOF), amounting to a strain isolation rate of 18.9% (Table 1). The minimal inhibitory concentration (MIC) breakpoints for amoxicillin, clarithromycin, metronidazole, tetracycline, and levofloxacin were 0.125, 0.5, 8, 1, and 1 mg/L, respectively. 

**Figure 1:**
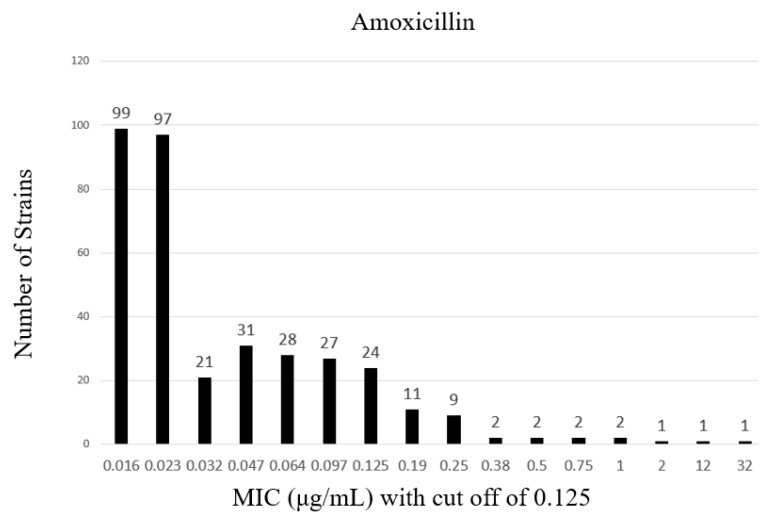
Distribution of amoxicillin resistance rates among clinical *Helicobacter pylori* strains.

**Figure 2:**
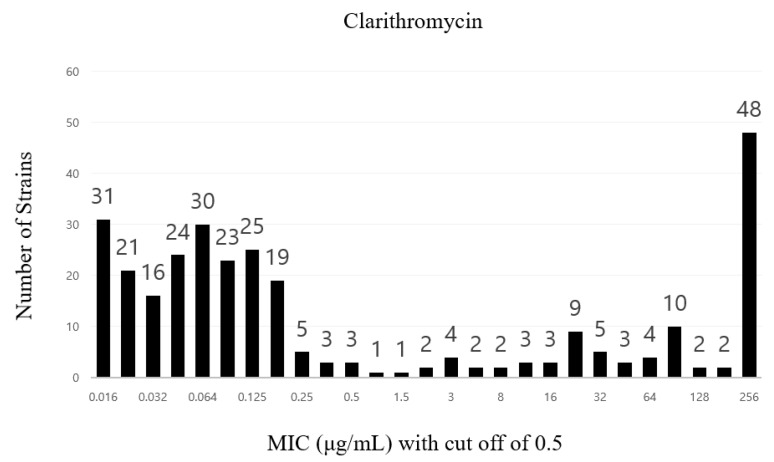
Distribution of clarithromycin resistance rates among clinical *Helicobacter pylori* strains.

Antibiotic susceptibility testing of the isolated *H. pylori* strains revealed that 31 were amoxicillin-resistant (antibiotic resistance rate of 8.7%) and 101 were clarithromycin-resistant (antibiotic resistance rate of 33.6%) (Figure 1 and Figure 2). 

The 9 amoxicillin-resistant strains (H-5, -6, -7, -8, -20, -303, -337, -341, and -346) were selected from the 31 resistant strains for WGS analysis, and the MICs for each strain were 0.19, 0.19, 12, 32, 0.25, 0.25, and 0.19 mg/L, respectively. 

The amoxicillin resistance genes used in the WGS analysis were penicillin-binding protein (*pbp*)1a, *pbp2*, Na+/H+ antiporters (*nhaC*), external beta-barrel protein HofH (*hofH*), external beta-barrel protein HofC (*hofC*), and efflux RND transporter transcriptase subunit HefC (*hefC*) (Table 2). 

The DNA sequences obtained using WGS analysis were compared and analyzed based on the ATCC 26695 reference strain. The WGS results of common amoxicillin-resistant genes’ SNPs are shown in Table 2. The amino acid mutations commonly observed in PBP1A were M17V, F125L, D479E, D535N, S589G, K648Q, R649K, and R656P, and the mutations observed in PBP2 were N16T, A26T, M97T, V218I, K240E, K359E, and S456H, N. NhaC exhibited amino acid mutations N53Y, H, I, V70I, G338E, and Y450H, HofH exhibited amino acid mutations S99G, I177V, K183H, N302D, L323R, F381L, T421S, N, Y436H, and D452N, HofC exhibited amino acid mutations A11T, A42S, A92V, G95S, K97R, Q175E, I197V, I206V, K221N, N222K, G230S, V242I, L349F, P382I, V383I, and T491N, and HefC exhibited amino acid mutations S316G and I361V. According to the analyzed strain, the mutations observed were A69V, V374L, S414R, T503I, A592D, and R435Q in PBP2 in the highly resistant strain H-8, with the highest minimum inhibitory concentration of 32 mg/L. 

Table 3 shows MLST results for clarithromycin-resistant and -sensitive strains. All isolated strains were newly found, which were inconsistent with the strains in the database. No substantial difference was observed between highly resistant strains (H-13, -33, -69, -189, -285, -313, and -325) and low-resistance strains (H-58, -64, -76, -103, -124, and -297). Similarly, there was no difference between resistant (H-13, -33, -58, -64, -69, -76, -103, -124, -189, -285, -297, -313, -325) and sensitive strains (H-15, -35, -114).

## 3. Discussion

*H. pylori* has reportedly infected approximately 4.4 billion people worldwide, and its prevalence varies from 18.9% to 87.7% in different regions [10]. *H. pylori* causes various diseases, such as chronic progressive gastritis, gastric ulcer, duodenal ulcer, and gastric cancer, and eradication of *H. pylori* reduces the incidence of gastric cancer [13]. Triple therapy with clarithromycin and PPIs is the treatment for *H. pylori* infection [14]. However, due to increasing clarithromycin resistance, the effectiveness of standard therapy is declining, and the World Health Organization is also aware of this problem [13]. Amoxicillin, another essential antibiotic in the *H. pylori* treatment regimen, also exhibits an increasing resistance rate of approximately 10% [13]. Selecting antibiotic combinations according to antibiotic susceptibility will enhance the success rate of *H. pylori* eradication [15]. However, *H. pylori* antibiotic susceptibility testing remains challenging due to the *H. pylori* cultivation difficulty and the time-consuming nature of *H. pylori* culture [16]. Clarithromycin resistance can be rapidly and readily identified using known resistance-associated SNPs through PCR. Using multiplex PCR (GenoType^®^ HelicoDR kit), the positive rate of clarithromycin resistance identification was 94.3%, which is higher than that of conventional methods, such as culture (77.1%) and histology (71.4%) [16]. A similar approach can be applied to identify amoxicillin resistance, another vital cause of *H. pylori* eradication failure. However, only a few SNPs associated with amoxicillin resistance have been reported because of a lack of molecular testing. The point mutation in PBP1A is the only reported SNP to date in amoxicillin resistance [17]. 

From the WGS analysis in this study, SNPs associated with amoxicillin resistance were identified in *pbp1a*, *pbp2*, *hofH*, and *hofC* [17]. F125L, D479E, D535N, S589G, K648Q, and R649K of *PBP1A* have been reported in previous studies [18,19,20]. This finding suggests that *pbp1* and *pbp2* SNPs induce antibiotic resistance by alternating the protein binding site, while *hofH* and *hofC* SNPs are associated with changes in the composition of the outer membrane.

In addition, 16 consecutive SNPs were observed in *hofC*. Therefore, it was considered a genetic change caused by a community acquisition mechanism rather than a SNP owing to a single mutation. This is because the strains used in the analysis were clinical strains isolated from patients treated at local hospitals. 

The H-8 strain, with the highest resistance at the minimum inhibitory concentration of 32 mg/L, presented mutations of A69V, V374L, S414R, T503I, and A592DP in PBP1A and R435Q in PBP2. The A69V, V374L, and S414R SNPs in PBP1A were identified in a previous study [6,21,22], but T503I and A592D in PBP1A, and R435Q in PBP2 were newly discovered SNPs from this study. In subsequent studies, additional amoxicillin-resistant clinical strains will need to be collected and analyzed to determine whether T503I, A592D, and R435Q in PBP1A are related to amoxicillin resistance, which has not been previously reported. 

This study had several limitations. Since *H. pylori* is difficult to culture under laboratory conditions, the isolation rate of *H. pylori* strains is only approximately 19% using culture. Therefore, cultural negativity does not ensure successful *H. pylori* eradication. M17V and R656P of PBP1A, which were first discovered in this study, require additional studies using other analytical methods. Furthermore, studies are required to analyze SNPs and MIC values with amoxicillin-resistance mechanisms. In the WGS, amoxicillin-sensitive *H. pylori* was not included in this analysis; therefore, it was difficult to ascertain whether these SNPs were directly related to resistance. 

In our study, the resistance rate of amoxicillin and tetracycline was low compared to previous studies. This low resistance rate may be attributed to regional, national, or racial differences. Antibiotic resistance rates vary in different regions. Our test results indicate characteristics of antibiotic resistance in South Korea, especially Seoul, the capital region. We can add MIC resistance data from previous *H. pylori* studies. 

Furthermore, we did not discuss tetracycline or levofloxacin in this study. We can discuss triple or *gyrA* mutations through additional analysis since we have WGS data. In addition, it would have been better to select amoxicillin-sensitive strains and compare them with amoxicillin-resistant strains to confirm that the SNPs we found contribute to amoxicillin resistance. However, we could only study strains with amoxicillin resistance and a reference ATCC 43504 strain due to funding limitations. 

In future studies, we can confirm that SNPs are associated with high amoxicillin resistance through mutagenesis. New mutations of PBP2 can be studied as well. Furthermore, amoxicillin-sensitive strains can be selected and compared. 

## 4. Materials and Methods

### 4.1. H. pylori Isolates and Culture Conditions

Clinical *H. pylori* strains were isolated from the gastric corpus and antrum specimens of patients who underwent an upper endoscopy at Gangnam Severance Hospital, Seoul, between March 2015 and June 2019. Detailed methods can be found in our institution’s published paper [14]. The specimens were transferred to the lab using a transport medium (Noble Biosciences Inc., Hwaseong, Republic of Korea). The patient samples were inoculated in egg yolk emulsion (EYE) agar (Yuhan LabTech, Seoul, Republic of Korea) and grown in a microaerophilic incubator (10% carbon dioxide, 5% oxygen, and 85% nitrogen at 37 °C) for 3–5 days. Although the EYE medium used in this study has a high contamination risk, it has a fast growth rate and easy visual identification due to the color change of the medium because it contains triphenyl tetrazolium chloride [23]. The grown strains were confirmed via visual inspection and identified using MALDI-TOF-MS (MALDI Biotyper™, Bruker, Billerica, MA, USA) and MBT Compass IVD software (Bruker, Middlesex County, MA, USA). *H. pylori* strain ATCC 43504 was used as the quality control. The *H. pylori* strain was inoculated into a Microbank™ (Pro-Lab Diagnostics, Richmond Hill, ON, Canada) and stored at −70 °C. 

### 4.2. H. pylori Isolates and Culture Conditions

Primary isolates of *H. pylori* colonies were sub-cultured for 2–4 days. Individual colonies were collected using aseptic needles and smeared into MSP 96 target-polished steel BC (Bruker, Middlesex County, MA, USA), and the colony was put into 1.5 µL of an α-cyano-4-hydroxycinnamic acid matrix (Bruker, Middlesex County, MA, USA). 

### 4.3. H. pylori Antibiotic Susceptibility Test

We performed an antimicrobial susceptibility test for amoxicillin on isolated *H. pylori* strains using the gradient diffusion method with E-test^®^ (bioMérieux, Marcy-l’Étoile, France). After inoculation at a concentration of 1.5 ± 0.5 McFarland using DensiCHEK-Plus (bioMérieux, Marcy-l’Étoile, France), the samples were placed in an Anoxomat™ AN2CTS system (MART Microbiology, B.V., Lichtenvoorde, Netherlands), replaced with microaerophilic conditions, and incubated at 37 °C for 48–72 h. According to the European Committee on Antimicrobial Susceptibility Testing (EUCAST) guidelines, strains were classified as resistant to amoxicillin when the MIC was >0.125 µg/mL. 

### 4.4. Whole-Genome Sequencing

For DNA extraction, a Gram-negative bacterial kit was used (GenElute™ Bacterial Genomic DNA Kit; G1N350, Sigma-Aldrich, St. Louis, MA, USA), according to the manufacturer’s instructions. DNA digestion was performed using g-tubes™ (Covaris, Woburn, MA, USA), so the average length of the extracted gDNA was 14–16 kb. A DNA library was prepared using the Preparing Multiplexed Microbial Libraries Using the SMRTbell^®^ Express Template Prep Kit 2.0 protocol method of the SMRTbell^®^ Express Template Prep Kit 2.0 (Pacific Biosciences, Menlo Park, CA, USA). The Microbial Multiplexing Calculator was used for pooling, and the size of 12.5–15 kb was selected. Quality control was performed using Genomic DNA ScreenTape Analysis and a Qubit™ dsDNA HS Assay Kit. For the final preparation, the volume, size, concentration, product version, and on-plate loading concentration of the sample were entered using the Sequel + New Calculation of SMRT^®^ Link Software (Pacific Biosciences). The SMRTbell™ template Sequel Binding and Internal Ctrl Kit 3.0 (Pacific Biosciences) was used for annealing and binding. WGS was performed with the SMRT Sequel^®^ II system (Pacific Biosciences). Movie time per SMRT cell (hours) was set to 10 h, and the pre-extension time was set to 2 h. The final sample (85 µL/well) was dispensed into a 96-well PCR plate (Pacific Biosciences), and each cell was sequenced for 12 h in the Sequel II System. The other experimental methods were performed according to the manufacturer’s instructions.

### 4.5. Multilocus Sequencing Typing (MLST)

A total of thirteen clarithromycin-resistant strains and three sensitive strains were selected from *H. pylori* clinical strains, incubated, and cultured on EYE agar plates. After culturing, *H. pylori* colonies were added to 1 mL of sterile water in an E-tube. After that, it was inactivated by heating at 95 °C in a heat block for 10 min. DNA was extracted from the inactivated samples using an InstaGene matrix (BIO-RAD), PCR was performed using a DNA Engine Tetrad 2 Peltier Thermal Cycler (BIO-RAD), and product purification was performed using a Multiscreen filter plate (Millipore Corp.). Sequencing was performed using an ABI PRISM 3730XL Analyzer (96 capillary type). The genes sequenced for MLST analysis were *atpA*, *efp*, *mutY*, *ppa*, *trpC*, *ureI*, and *yphC*, and allele and sequence types were determined. 

The primer sequences of *atpA* were 5′-GGACTAGCGTTAAACGCACG-3′ (forward primer) and 5′-CTTGAAACCGACAAGCCCAC-3′ (reverse primer), and the size of the PCR product was 627 base pairs. The primer sequences of *efp* were 5′-GGCAATTTGGATGAGCGAGCTC-3′ (forward primer) and 5′-CTTCACCTTTTCAAGATACTC-3′ (reverse primer), and the size of the PCR product was 410 base pairs. The primer sequences of *mutY* were 5′-TGGTTGTAGYTGGAAACTTTACAC-3′ (forward primer) and 5′-CTTAAGCGTGTGTYTTTCTAGG-3′ (reverse primer), and the size of the PCR product was 420 base pairs. The primer sequences of *ppa* were 5′-GGAGATTGCAATGAATTTAGA-3′ (forward primer) and 5′-GTGGGGTTAARATCGTTAAATTG-3′ (reverse primer), and the size of the PCR product was 396 base pairs. The primer sequences of *trpC* were 5′-TAGAATGCAAAAAAGCATCGCCCTC-3′ (forward primer) and 5′-TAAGCCCGCACACTTTATTTTCGCC-3′ (reverse primer), and the size of the PCR product was 627 base pairs. The primer sequences of *ureI* were 5′-AGGTTATTCGTAAGGTGCG-3′ (forward primer) and 5′-GTTTAAATCCCTTAGATTGCC-3′ (reverse primer), and the size of the PCR product was 585 base pairs. The primer sequences of *yphC* were 5′-CACGCCTATTTTTTTGACTAAAAAC-3′ (forward primer) and 5′-CATTYACCCTCCCAATGATGC-3′ (reverse primer), and the size of the PCR product was 510 base pairs. All strains were new strain types that did not match the existing database. No significant difference was confirmed between high resistance (H-13, -33, -69, -189, -285, -313, and -325) and low resistance (H-58, -64, -76, -103, -124, and -297). In addition, there was no significant difference between resistant (H-13, -33, -58, -64, -69, -76, -103, -124, -189, -285, -297, -313, and -325) and susceptible (H-15, -35, and -114) strains.

## 5. Conclusions

We verified the previously reported SNPs related to amoxicillin resistance using WGS and discovered additional SNPs likely associated with amoxicillin resistance. The discovery of SNPs related to amoxicillin resistance will help understand treatment failures and guide antibiotic selection for *H. pylori* eradication. 

## Figures and Tables

**Table 1:** Antibiotic susceptibility test for *Helicobacter pylori* identified from clinical specimens.

No. of Total Clinical Specimens			1952
No. of *H. pylori* Identified by MALDI-TOF-MS			368
AST Results Using an E-Test	Antibiotic Type	MIC Breakpoint (mg/L)	No. of Strains
	Amoxicillin	>0.125	31
	Clarithromycin	>0.5	101
	Metronidazole	>8	68
	Tetracycline	>1	20
	Levofloxacin	>1	114

AST, antimicrobial susceptibility test; MALDI-TOF; matrix-assisted laser desorption/ionization time-of-flight mass spectrometer, E-test; Epsilometer test; MIC, minimum inhibitory concentration; EUCAST, European Committee on Antimicrobial Susceptibility Testing. The MIC breakpoint was determined using EUCAST version 12.0.

**Table 2:** WGS analysis results of amoxicillin-resistant *Helicobacter pylori*.

Gene ^1^	Common SNPs in Nine Amoxicillin-Resistant Strains
*pbp1a* (8)	M17V, F125L, D479E, D535N, S589G, K648Q, R649K, R656P
*pbp2* (7)	N16T, A26T, M97T, V218I, K240E, K359E, S456H, N
*nhaC* (4)	N53Y, H, I, V70I, G338E, Y450H
*hofH* (9)	S99G, I177V, K183H, N302D, L323R, F381L, T421S, N, Y436H, D452N
*hofC* (16)	A11T, A42S, A92V, G95S, K97R, Q175E, I197V, I206V, K221N, N222K, G230S, V242I, L349F, P382I, V383I, T491N
*hefC* (2)	S316G, I361V

^1^ Common amoxicillin-resistant genes. The SMRT Sequel^®^ II system (Pacific Biosciences) and the SnapGene^®^ Viewer program were used for this result. A: adenine, D: aspartic acid, E: glutamic acid, F: phenylalanine, G: glycine, H: histidine, I: isoleucine, K: lysine, L: leucine, M: methionine, N: asparagine, P: proline, Q: glutamine, R: arginine, S: serine, T: threonine, V: valine, and Y: tyrosine. *1pbp1a*, penicillin-binding protein 1A gene; *pbp2*, penicillin-binding protein 2 gene; *nhaC*, Na+:H+ antiporter gene; *hofH*, external beta-barrel protein gene; *hofC*, external beta-barrel protein gene; *hefC*, efflux RND transporter transcriptase subunit gene; SNP, single-nucleotide polymorphism; WGS, whole-genome sequencing.

**Table 3:** MLST results for clarithromycin-resistant and -sensitive strains.

*H. pylori* Strain	MIC90 (ug/mL) ^a^	ST (Strain Type)	Allele						
			atpA	epf	mutY	ppa	trpC	ure1	yphC
H-13	<256	-	1400 ^b^	1893 ^b^	472 ^c^	1822 ^b^	426 ^b^	3013 ^b^	72 ^c^
H-33	<256	-	825 ^c^	2423 ^b^	3149 ^b^	1957 ^b^	735 ^c^	44 ^b^	1932 ^b^
H-58	8	-	2894 ^b^	1815 ^b^	1970 ^b^	426	54 ^b^	54 ^c^	1971 ^b^
H-64	16	-	1216 ^b^	2421 ^b^	1968 ^c^	1517	3015 ^b^	1979 ^b^	3037 ^b^
H-69	<256	-	2720 ^b^	2703 ^b^	1979 ^c^	64 ^b^	3236 ^c^	2096 ^b^	2678 ^b^
H-76	2	-	2894 ^b^	1815 ^b^	1970 ^b^	426	54 ^b^	54 ^c^	1971 ^b^
H-103	48	-	2750 ^c^	1278 ^b^	493 ^c^	1066 ^b^	62 ^c^	2804 ^b^	3076 ^b^
H-124	3	-	50 ^b^	2331 ^b^	725 ^b^	2754 ^b^	50 ^b^	2804 ^b^	3295 ^c^
H-189	<256	-	2750 ^c^	499 ^b^	2360 ^b^	1125 ^b^	1526 ^c^	2804 ^b^	3076 ^b^
H-285	192	-	3114 ^b^	1167 ^b^	452 ^b^	51	2976	905 ^b^	1986 ^b^
H-297	6	-	53 ^b^	424 ^b^	3149 ^b^	168 ^b^	967 ^b^	2000 ^b^	1962 ^b^
H-313	<256	-	50 ^b^	2930 ^b^	50 ^b^	64 ^b^	3254 ^c^	2829 ^b^	919 ^b^
H-325	96	-	2753 ^b^	2714 ^b^	1355 ^b^	1917 ^b^	967 ^b^	2103 ^b^	34 ^c^
H-15	0.016	-	1096 ^b^	1908 ^b^	725 ^c^	940 ^b^	50 ^b^	424 ^b^	515 ^b^
H-35	0.047	-	3114 ^b^	704 ^b^	3188 ^b^	1066 ^b^	1241 ^b^	883 ^b^	2076 ^c^
H-114	0.016	-	1870 ^b^	701 ^b^	2969 ^c^	459 ^b^	50 ^c^	3254 ^b^	373 ^b^

^a^ followed EUCAST Clinical Breakpoint Tables v. 11.0; ^b^ 1~5 missmaching Number of MLST Database; ^c^ 6~15 missmaching Number of MLST Database.

## Data Availability

The data presented in the study are available upon request.
